# Morphological and phylogenetic characterization of three new *Cortinarius* (Cortinariaceae, Agaricales) species in Yunnan, China

**DOI:** 10.3897/mycokeys.130.170601

**Published:** 2026-03-27

**Authors:** Tai-Shun Li, Hong-Bin Guan, Yan-Yan Yang, Fang Wu, Qi Zhao, Ying Zhang

**Affiliations:** 1 Key Laboratory of Forest Disaster Warning and Control in Yunnan Province, Southwest Forestry University, Kunming, Yunnan 650224, China Beijing Forestry University Beijing China; 2 State Key Laboratory of Phytochemistry and Natural Medicines, Kunming Institute of Botany, Chinese Academy of Sciences, Kunming, Yunnan 650201, China Kunming Institute of Botany, Chinese Academy of Sciences Kunming China; 3 State Key Laboratory of Efficient Production of Forest Resources, School of Ecology and Nature Conservation, Beijing Forestry University, Beijing 100083, China Southwest Forestry University Kunming China

**Keywords:** Morphology, new taxa, sect. *Delibuti*, sect. *Defibulati*

## Abstract

Through comprehensive morphological and phylogenetic investigations (ITS and nrLSU), we have delineated three novel species within *Cortinarius*, as derived from specimens collected in Yunnan Province, China. *Cortinarius mucobrunneus* is distinguished by its viscid basidiomata, dark brown pileus adorned with translucent stripes, cinnamon to brown lamellae, white stipe leaving a cinnamon ring on the upper part, and elliptical to amygdaliform, strongly verrucose basidiospores measuring 13.5–16 × 8–10 μm. *Cortinarius rugosiviscidus* is characterized by sticky basidiomata, a light brown pileus with wrinkled surface, brown lamellae, light brown stipe, and elliptical to amygdaliform, moderately verrucose basidiospores measuring 11.5–15 × 7.5–9.5 μm. *Cortinarius saloroides* is characterized by its violet pileus with fine fibrous scales, mauve to light brown lamellae, bluish violet stipe leaving a light brown ring on the upper part, and subspherical, broadly elliptical to elliptical, moderately verrucose basidiospores measuring 7–8.5 × 5.5–6.5 μm. Differences between the new species and morphologically similar and phylogenetically related species are discussed.

## Introduction

*Cortinarius* (Pers.) Gray is one of the most species-rich genera in the phylum Basidiomycota R.T. Moore. More than 5,881 records of *Cortinarius* are listed in Index Fungorum (https://www.indexfungorum.org/Names/Names.asp, accessed on 25 August 2025). Its taxonomic history can be traced back to Persoon (1801), who first described it as a section within the genus *Agaricus* L., and later Gray (1821) elevated it to the rank of an independent genus. This genus exhibits distinct morphological characteristics, which comprise a gleaming luster on the surface of the fruiting body, a cobweb-like veil present between the pileus and stipe during its juvenile stage, and mature basidiospores that range from rusty brown to brown, characterized by a warty surface texture (Moser and Horak 1975; Singer 1986). While macroscopic morphology is generally easily identifiable, the subtle morphological differences among species have posed significant challenges for traditional classification. In recent years, the meticulous investigation of morphology, coupled with molecular phylogenetics, has emerged as a pivotal approach for reevaluating the taxonomic framework of this genus (Seidl 2000; Soop et al. 2019; Xie et al. 2021a, 2025; Long et al. 2024).

*Cortinarius* exhibits a broad ecological adaptability and forms ectomycorrhizal symbiotic relationships with a wide range of woody and herbaceous plants, including Betulaceae Gray, Cyperaceae Juss., Fagaceae Dumort., Nothofagaceae Kuprian., Orchidaceae Juss., Pinaceae Spreng. ex F.Rudolphi, and Salicaceae Mirb. (Harrington and Mitchell 2002; Jacquemyn et al. 2010; Tedersoo et al. 2011; Harrower et al. 2015; Thoen et al. 2019). It plays a key role in forest ecosystems, regulating nutrient cycling and community stability. Some species, such as *Cortinarius armillatus* (Fr.) Fr., *C. bovinus* Fr., and *C. emodensis* Berk. possess both edible and medicinal value. However, a few species, including *C. gentilis* (Fr.) Fr., *C. orellanus* Fr., and *C. rubellus* Cooke, contain the toxin orellanine, which can cause renal failure-type poisoning upon accidental ingestion (Bau et al. 2024).

In the classification system of *Cortinarius*, sect. *Delibuti* (Fr.) Henn. and sect. *Defibulati* M.M. Moser are two important groups. Early classifications were based on the mucus characteristics of the pileus and stipe, placing them within *Cortinarius* subg. *Myxacium* (Fr.) Trog (Trog 1844; Earle 1902; Singer 1986; Seidl 2000; Garnica et al. 2005). However, molecular systematic studies by Soop et al. (2019) showed that sect. *Delibuti* is more appropriately placed within the *Anomaloid* sections. Some European scholars regard sect. *Defibulati* as a subsect of sect. *Colliniti* (Brandrud et al. 1990, 1992; Bendiksen et al. 1993). Seidl (2000) pointed out through phylogenetic analysis that sect. *Defibulati* and sect. *Myxacium* are closely related and monophyletic, while sect. *Delibuti* is on a more distant branch in the phylogenetic tree.

During a survey of fungal diversity in the Qinghai-Xizang Plateau and its surrounding areas in China, six *Cortinarius* specimens characterized by a viscid pileus were collected (Wang et al. 2025). A comprehensive morphological assessment, supplemented by detailed illustrations and descriptions, was conducted in conjunction with multi-locus phylogenetic analyses to elucidate the taxonomic status of these novel populations. Sequence data derived from the ITS and nrLSU regions revealed that these strains consistently clustered into distinct clades with robust statistical support. Morphological evaluations indicated unique distinguishing features among our specimens. Consequently, we propose three new taxa, accompanied by their full descriptions, illustrative representations, and detailed phylogenetic relationships.

## Materials and methods

### Sample collection

Six specimens of *Cortinarius* were collected from *Abies* forests in Yunnan Province, China, between August 2017 and August 2024. During the collection process, habitat photos were taken by digital cameras (Canon, Japan), and relevant information such as the collection location, time, altitude, longitude, and latitude was recorded (Rathnayaka et al. 2024). The specimens were described in detail in terms of macroscopic morphology, including the pileus, context, lamellae, and stipe. Some fresh specimens were dried using silica gel desiccant for DNA extraction, while the remaining specimens were dried in a food dryer at 40 °C (Hu et al. 2022). The newly collected specimens were deposited in the Herbarium of Cryptogams of Kunming Institute of Botany, Chinese Academy of Sciences (KUN-HKAS), China.

### Morphological observation

Samples were observed and examined using the method described by Zhao et al. (2015). Macroscopic and microscopic features were examined using a stereomicroscope (SteREO Discovery.V12, Carl Zeiss Microscopy GmBH, Germany), and microphotographs were taken using a compound microscope (Nikon ECLIPSE 80i, Nikon, Japan) fitted with a NikonDS-Ri2 digital camera (Nikon, Japan). Color codes indicated in the descriptions were based on Kornerup and Wanscher (1981). Dried specimens were sectioned and mounted in a 5% KOH solution and Melzer’s reagent, using 5% Lugol reagent for observing iodine reaction. Congo red reagent was used for staining to facilitate observation of the microscopic structure. In order to observe the ornamentation on the surface of basidiospores, some hymenophoral fragments were cut from the dry specimens, fixed on aluminum stubs, coated with gold-palladium, and then observed under a ZEISS Sigma 300 scanning electron microscope (SEM) at the Kunming Institute of Botany.

Microstructure measurements were performed using Image Frame Work v.0.9.7. Measurements were given as (a–) b–c (–d), where a denoted the minimum value, d was the maximum value, and b–c was the 90% confidence interval. The measured number of basidiospores (n), basidiomata (m), and specimens (p) was denoted as [n/m/p]. The Q value indicated the length to width ratio of the basidiospores, while **Q** indicated the average of the length to width ratios (Q) of all ascospores ± standard deviation. The illustrations were done using Adobe Photoshop 2020 (Adobe Systems, USA).

### DNA extraction, PCR amplification, and sequencing

DNA was extracted from fruiting bodies of *Cortinarius* directly using Trelief^TM^ Fungal Genomic DNA Extraction Kit (Tsingke Biotechnology Co., Ltd., Beijing, China). Polymerase chain reaction (PCR) was used to amplify the nuclear ribosomal internal transcribed spacer (ITS) and the large subunit of the nuclear ribosomal RNA (nrLSU). Universal primer pairs ITS5/ITS4 (White et al. 1990) and LR0R/LR5 (Vilgalys and Hester 1990) were used to amplify the ITS and nrLSU loci, respectively. The PCR conditions are as follows: an initial denaturation at 95 °C for 5 min, followed by 35 cycles of denaturation at 95 °C for 30 s, annealing at 53 °C for 20 s, and extension at 72 °C for 30 s, followed by a final extension at 72 °C for 10 min. PCR products were sent to Tsingke Company in Beijing, China, for sequencing.

### Phylogenetic analyses

The newly generated sequences were checked for ambiguous bases and assembled using DNASTAR Lasergene SeqMan Pro v.7.1.0. This study used new sequencing data and reliable data from the literature to construct a phylogenetic tree (Table 1), mainly referring to the research results of Soop et al. (2021), Xie et al. (2021a), Long et al. (2024), and Xie et al. (2025). Multiple sequence alignments were aligned in MAFFT v.7 (https://mafft.cbrc.jp/alignment/server/index.html, Katoh and Standley 2013; Katoh et al. 2019), and trimmed using TrimAl v.1.3 (Capella-Gutiérrez et al. 2009). The ‘gapthreshold’ was set to 0.5 for both ITS and nrLSU regions. The trimmed sequences were sequentially assembled into a combined dataset using SequenceMatrix V.1.8 software (Vaidya et al. 2011).

**Table 1.** Names, voucher numbers, countries, references, and corresponding GenBank accession numbers of the taxa used in this study. Names in red background indicate newly described species in this study. All type specimens are highlighted with a “T”. The symbol “–” denotes no available data.

Species	Voucher	Locality	GenBank Accession No.		References
			ITS	nrLSU	
*Cortinarius acutovelatus*	F16388	Canada	FJ039609	–	Harrower et al. (2011)
*C. albocyaneus* **T**	S:CFP1177	Sweden	KX302206	–	Dima et al. (2016)
*C. alboluteus* **T**	DAOM R. Lebeuf HRL2038	USA	NR_157977	–	Genbank
*C. alpinus*	HMJAU44407	China, Neimenggu	MW911727	–	Xie et al. (2021a)
*C. anomalus*	NL-5414	Hungary	MZ663777	–	Dima et al. (2021)
*C. anomalus* **T**	S:CFP1154	Sweden	KX302224	–	Dima et al. (2016)
*C. basipurpureus*	PERTH 04259629	Australia	AY669607	AY669607	Garnica et al. (2005)
*C. bolaris*	TUB 0118524	Germany	AY669596	AY669596	Garnica et al. (2005)
*C. brunneoalbus* **T**	TN09-075	USA	NR_153040	–	Ariyawansa et al. (2015)
*C. calaisopus* **T**	PDD:94050	New Zealand	NR_157880	NG_068868	Genbank
*C. camphoratus*	DAVFP26155	Canada	EU821659	EU821659	Harrower et al. (2011)
*C. camphoratus*	SMI193	Canada	FJ039626	FJ039626	Harrower et al. (2011)
*C. carneoroseus*	EN76 (CORD)	Argentina	JX983157	–	GenBank
*C. cf. tibeticisalor*	HMJAU58970	China, Xizang	PQ278654	PQ489285	Xie et al. (2025)
*C. collinitus*	IB19940257	Sweden	AY033096	AY033138	Peintner et al. (2002)
*C. corpulentus* **T**	DBB49315	USA	OP874939	–	GenBank
*C. costaricensis* **T**	JFA 11904	Germany	EF420147	–	Ammirati et al. (2007)
*C. camphoratus*	DAVFP26155	Canada	EU821659	EU821659	Harrower et al. (2011)
*C. cuphocyboides*	CO1018	Germany	AY669625	AY669625	Garnica et al. (2005)
*C. cypripedi* **T**	PDD107723	New Zealand	KT875199	–	Soop (2016)
*C. cystidiocatenatus*	HO A20518A6	Germany	AY669651	AY669651	Garnica et al. (2005)
*C. delibutus*	F17048	Canada	FJ717515	FJ717515	Harrower et al. (2011)
*C. delibutus*	SAT01-301-12	USA	FJ717513	FJ717513	Harrower et al. (2011)
*C. dryadicola*	HMJAU58991	China, Yunnan	PQ278684	PQ489266	Xie et al. (2025)
*C. dryosalor*	AB05-10-302	France	OM964840	–	GenBank
*C. dryosalor* **T**	FA4700	Spain	OM964838	ON032996	GenBank
*C. durifoliorum* **T**	PDD:101829	New Zealand	KJ635210	MW263597	Soop et al. (2018)
*C. eunomalus*	PDD 107706	New Zealand	KT875201	MW263617	GenBank
*C. fibrillososalor*	MHHNU 32070	China, Hunan	OR660685	OR647503	Long et al. (2024)
*C. fibrillososalor* **T**	MHHNU 32494	China, Hunan	OR647481	OR647506	Long et al. (2024)
*C. gymnocephalus* **T**	CO1334	Germany	AY669629	AY669629	Garnica et al. (2005)
*C. illibatus*	HMJAU48760	China, Heilongjiang	MW911735	OP620668	Xie et al. (2021a)
*C. illibatus*	OS574	Norway	KC842441	KC842511	Stensrud et al. (2014)
*C. illitus* **T**	IB1963414	Argentina	AF389128	AF388751	Peintner et al. (2004)
*C. illumines* **T**	S:F44877	Sweden	KP866156	–	Niskanen et al. (2015)
*C. iodes*	MQ19-CMMF003109	Canada	MN751331	–	GenBank
*C. khinganensis* **T**	HMJAU44507	China, Neimenggu	MT299952	OM001525	Xie et al. (2021b)
*C. lewisii*	iNAT130719864	USA	OP643406	–	GenBank
*C. lilacinicarpus* **T**	HMJAU58977	China, Fujian	PQ278675	PQ489270	Xie et al. (2025)
*C. lilacinicarpus*	KUN-HKAS81961	China, Guangdong	PQ278676	PQ489271	Xie et al. (2025)
*C. microglobisporus* **T**	IB20110123	Italy	NR_153027	–	Peintner et al. (2014)
*C. mucifluus*	TAAM128775	Estonia	UDB015965	–	Unite
*C. mucobrunneus*	HKAS 149456	China, Yunnan	PV992732	–	This study
*C. mucobrunneus* **T**	HKAS 149459	China, Yunnan	PV992733	PV992752	This study
*C. obtusus*	SAT00-298-30	USA	FJ717550	FJ717550	Harrower et al. (2011)
*C. ochroglutinosus* **T**	H7000818	Canada	NR_173073	–	Liimatainen and Niskanen 2021
*C. parviviscidus*	WTU-F-78349	Guyana	OR147297	PP468371	Siegel et al. (2024)
*C. paveleckii* **T**	Trappe 7962	USA	AF325564	–	Peintner et al. (2004)
*C. paveleckii*	OSC 62148	USA	KT968601	KU321878	GenBank
*C. phlegmophorus* **T**	Typus-M3	India	AY083186	–	Peintner et al. (2003)
*C. porphyroideus*	OTA:61406	New Zealand	JX178612	KT334150	Teasdale et al. (2013)
*C. pseudocandelaris*	UBC F17165 OC93	Canada	GQ159908	GQ159908	Harrower et al. (2011)
*C. pseudosalor*	MHHNU 32148	China, Hubei	OR660688	OR647505	Long et al. (2024)
*C. pseudosalor* **T**	MHHNU 32082	China, Hubei	OR660686	OR647504	Long et al. (2024)
*C. putorius* **T**	H T. Niskanen 07-411	USA	NR_153038	–	Ariyawansa et al. (2015)
*C. pyrenaicus*	JB-8573/15	Spain	KX239900	–	GenBank
*C. rattinoides* **T**	PDD:88283	New Zealand	JX000375	NG_064341	GenBank
*C. rotundisporus*	PERTH05255074	Australia	AY669612	AY669612	Garnica et al. (2005)
*C. rotundisporus*	G12	Australia	AF136738	–	Sawyer et al. (1999)
*C. rugosiviscidus*	HKAS 149457	China, Yunnan	PV992734	PV992753	This study
*C. rugosiviscidus* **T**	HKAS 149458	China, Yunnan	PV992735	PV992754	This study
*C. salor*	TUB011838	Germany	AY669592	AY669592	Garnica et al. (2005)
*C. seidliae* **T**	H:T. Niskanen 09-132	Finland	KR011125	KR011125	Ariyawansa et al. (2015)
*C. septentrionalis*	ARANFungi03516	Sweden	KX239915	–	GenBank
*C. sinocalaisopus*	HMJAU58978	China, Fujian	PQ278677	PQ489277	Xie et al. (2025)
*C. sinocalaisopus* **T**	HMJAU58984	China, Fujian	PQ278683	–	Xie et al. (2025)
*C. sinosalor* **T**	MHHNU30649	China, Hunan	PQ278661	PQ489281	Xie et al. (2025)
*C. sinosalor*	HMJAU58971	China, Zhejiang	PQ278662	PQ489282	Xie et al. (2025)
*C. spilomeus* **T**	S:CFP1137	Sweden	KX302267	–	Dima et al. (2016)
*C. stillatitus*	TUB 011587	Germany	AY669589	AY669589	Garnica et al. (2005)
*C. subiodes* **T**	K:M 001434108	USA	NR_190944	–	Liu et al. (2024)
*C. subiodes*	K:M 001434110	USA	ON843414	–	Liu et al. (2024)
*C. subsalor*	HMJAU48759	China, Zhejiang	MW911734	OP620670	Xie et al. (2021a)
*C. subtropicus*	MHHNU 31981	China, Hunan	OR660687	OR647502	Long et al. (2024)
*C. subtropicus* **T**	MHHNU 33533	China, Hunan	OR647488	OR647508	Long et al. (2024)
*C. subviolaceus*	AF69	Australia	DQ328114	–	GenBank
*C. suecicolor* **T**	PDD74698	New Zealand	JX000360	JX000391	GenBank
*C. tabularis* **T**	S:CFP949	Sweden	KX302275	–	Dima et al. (2016)
*C. tasmacamphoratus*	HO A20606A0	Tasmania	AY669633	AY669633	Garnica et al. (2005)
*C. tessiae*	PDD94054	New Zealand	JQ287698	–	GenBank
*C. tessiae*	PDD72611	New Zealand	HM060317	HM060316	GenBank
*C. tianbaoyanensis*	HMJAU58987	China, Fujian	PQ278685	PQ489283	Xie et al. (2025)
*C. tibeticisalor*	HMJAU48763	China, Xizang	MW911730	–	Xie et al. (2021a)
*C. tibeticisalor* **T**	HMJAU48764	China, Xizang	MW911729	OP620669	Xie et al. (2021a)
*C. saloroides*	HKAS 149460	China, Yunnan	PV992736	PV992755	This study
*C. saloroides* **T**	HKAS 149461	China, Yunnan	PV992737	PV992756	This study
*C. vanduzerensis*	VMS28	Canada	FJ717562	FJ717562	Harrower et al. (2011)
*C. viridipileatus* **T**	OTA64087	New Zealand	MK546593	MK546596	Nilsen et al. (2021)
*C. vividus* **T**	HMJAU58972	China, Chongqing	PQ278664	PQ489289	Xie et al. (2025)
*C. vividus*	HMJAU58974	China, Zhejiang	PQ278666	PQ489287	Xie et al. (2025)
*Cortinarius* sp.	SWUBC500	Canada	DQ481723	–	GenBank
*Phlegmacium boreicyanites* **T**	S:CFP931	Sweden	NR_130214	–	Liimatainen et al. (2014)
*Ph. cyanites* **T**	UPS A. Taylor 2005069	Sweden	NR_130233	–	Liimatainen et al. (2014)

Maximum likelihood (ML) analysis was performed in the CIPRES Science Gateway (http://www.phylo.org/) platform using RAxML-HPC2 v.8.2.12 (Miller et al. 2010). The substitution model for ML analysis of all gene regions was GTR+I+G, with 1,000 rapid bootstrap replicates. Bayesian inference (BI) analysis was performed using MrBayes v.3.2, using Markov Chain Monte Carlo Sampling (MCMC) to calculate a posterior probability (PP). MrModeltest v.2.3 (Nylander et al. 2004) was used to determine the best-fit model for the evolution of each gene using the Akaike information criterion, and GTR+I+G substitutions were ultimately selected as the appropriate models for ITS and nrLSU. Four simultaneous Markov chains operated simultaneously for 1195000 generations, and samples were taken every 100^th^ generation. The first 25% of trees were discarded due to aging, and convergence was determined when the standard deviation of the split frequencies reached 0.01 and the effective sample size (ESS) > 200. The phylogenetic tree was visualized using FigTree v.1.4.4 and reorganized in Adobe Illustrator CC 2018 (Adobe Systems, USA).

## Results

### Molecular analyses

**Figure 1.**
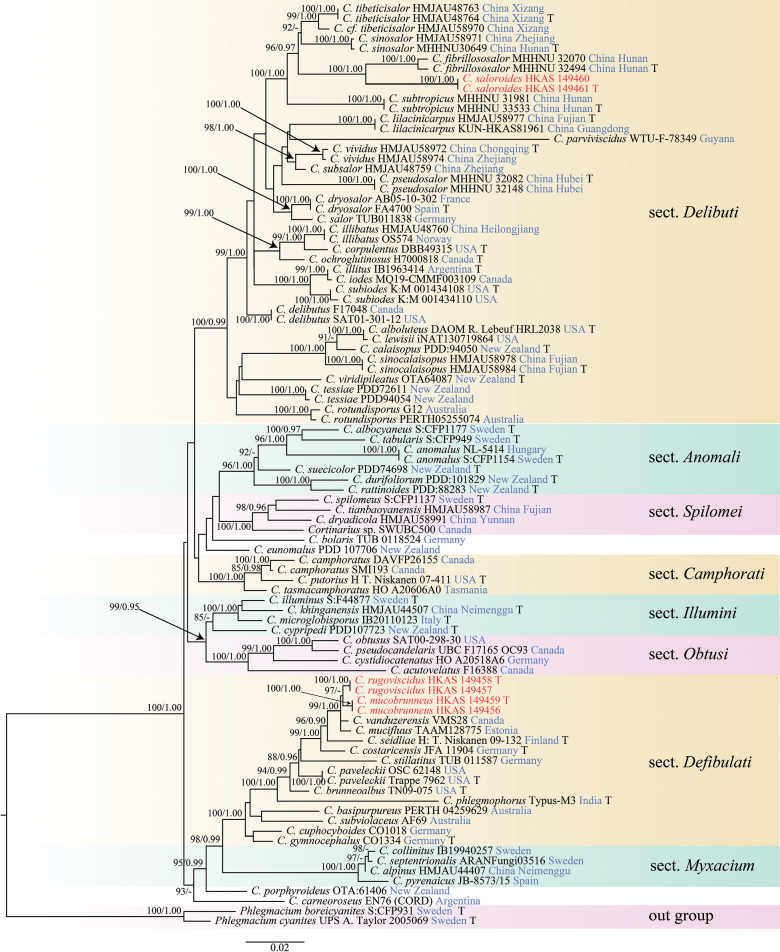
Maximum likelihood phylogenetic tree generated from the combined ITS and nrLSU sequenced dataset. Maximum likelihood bootstrap (ML-BP) ≥ 85% and Bayesian posterior probabilities (BI-PP) ≥ 0.90 are indicated above the nodes. “-” indicates that the support value was less than the respective threshold. The specimen vouchers and country are indicated after the species names. New samples collected in this study are indicated in red. “T” indicates that the type specimens.

The concatenated matrix comprised 150 sequences (93 in ITS and 57 in nrLSU), representing 58 samples. Following Xie et al. (2021a), *Phlegmacium boreicyanites* (S: CFP931) and *P. cyanites* (UPS: A. Taylor 2005069) were treated as the outgroup taxa. The alignment contained 1,548 characters, including gaps (ITS: 1–647, nrLSU: 648–1,548), of which 1,061 characters were constant, 365 characters were parsimony-informative, and 122 were singleton sites. The estimated base frequencies were as follows: A = 0.258, C = 0.190, G = 0.244, T = 0.308, substitution rates AC = 1.81594, AG = 5.60442, AT = 1.81594, CG = 1.00000, CT = 8.94348, GT = 1.00000; and gamma distribution shape parameter α = 0.675. The ML and BI analyses showed similar topology, and the topology from the ML analysis was presented along with statistical values from the MLBS/BIPP algorithms (Fig. 1).

Phylogenetic analysis showed that all species formed separate monophyletic lineages. Our three new species are clustered in two different sections. *Cortinarius saloroides* clustered in sect. *Delibuti*, while *C. rugosiviscidus* and *C. mucobrunneus* clustered in sect. *Defibulati*. In sect. *Delibuti*, *Cortinarius saloroides*, and *C. fibrillososalor* were sister lineages (BP = 100%, PP = 1.00), forming a clade with *C. subtropicus* and *C. tibeticisalor*, which were only distributed in East Asia. In sect. *Defibulati*, *Cortinarius rugosiviscidus*, and *C. mucobrunneus* were sister lineages (BP = 97%), and had a close relationship with *C. vanduzerensis* and *C. mucifluus* (BP = 99%, PP = 1.00).

### Taxonomy

#### Cortinarius mucobrunneus

Taxon classificationFungiAgaricalesCortinariaceae

T.S. Li, Y. Zhang & Q. Zhao sp. nov

15BEB658-3D43-5BF2-A0FE-C479034C6A5C

860475

**Figure 2.**
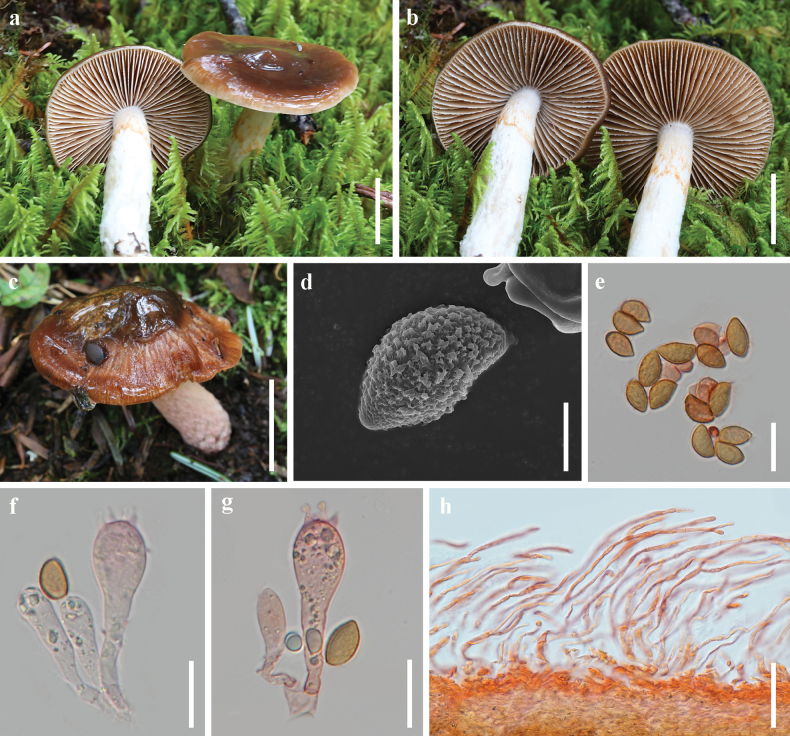
*Cortinarius mucobrunneus*. a–c. Basidiomata (a, b. HKAS 149459; c. HKAS 149456); d. Scanning electron micrograph of basidiospore; e. Basidiospores; f, g. Basidia; h. Pileipellis. Scale bars: 2 cm (a–c); 5 μm (d); 20 μm (e–g); 50 μm (h).

Fig. 2 Chinese name: 黏褐丝膜菌 (nian he si mo jun)

##### Etymology.

mucobrunneus refers to the brown pileus with mucus on the surface.

##### Holotype.

China • Yunnan Province: Lijiang City, Laojunshan Nature Reserve, at 26.631426°N, 99.719085°E, alt. 3960 m, in *Abies* forest, 26 Aug. 2018, LJ1678 (HKAS 149459).

##### Diagnosis.

Similar to *Cortinarius vanduzerensis* in the sticky pileus surface, but differing in the larger basidiospores.

##### Macrostructures.

***Basidiomata*** medium-sized. ***Pileus*** 40–60 mm diam., conical to campanulate, becoming applanate and umbonate with age; surface viscid, dark brown (7F8) to liver brown (8F8); margin tile red (7D8) to platinum blonde (4B3), undulate, translucent radially striate; context 4–5 mm thick at mid-radius, white. ***Lamellae*** adnate to adnexed, 3–4 mm broad, moderately crowded, cinnamon (6D4) to brown (6E7); edge serrate. ***Stipe*** 50–80 × 7–12 mm, cylindrical, usually tapering upwards; surface viscid, white, leaving a cinnamon (6D5) ring on the upper part of the stipe. Basal mycelium white. ***Odour*** not distinctive.

##### Microstructures.

***Basidiospores*** [100/2/2] (13–) 13.5–16 (–17) × 8–10 (–10.5) μm [Q = (1.42) 1.52–1.91 (–2), **Q** = 1.66 ± 0.09], elliptical to amygdaliform, cinnamon in KOH, strongly verrucose. ***Basidia*** 40–57 × 14–17 μm, clavate, 4-spored. ***Lamella trama*** hyphae 4–16 μm diam., smooth, colourless. Lamellar edges fertile. ***Pileipellis*** duplex: epicutis strongly gelatinous, 110–230 μm thick, composed of flat, loosely arranged, long-celled, colourless hyphae 4–10 μm diam.; hypocutis 35–70 μm thick, composed of cylindrical to elliptical, strongly interwoven, colourless hyphae 4–12 μm diam., some hyphae with spiral or spotted encrustations. ***Stipe hyphae*** 4–10 μm diam., colourless, smooth. ***Clamp connections*** absent.

##### Habitat.

Mycorrhizal, gregarious in coniferous forests dominated by *Picea* spp. and *Abies* spp. trees.

##### Distribution.

Known from the southern Yunnan Province, China.

##### Additional material examined.

China • Yunnan Province: Lijiang City, Laojunshan Nature Reserve, at 26.631426°N, 99.719085°E, alt. 3960 m, in *Abies* forest, 16 Aug. 2017, LJ981 (HKAS 149456).

##### Notes.

*Cortinarius mucobrunneus* is characterized by its victid basidiomata, dark brown pileus with translucent stripes, cinnamon to brown lamellae, white stipe leaving a cinnamon ring on the upper part, and elliptical to amygdaliform, strongly warty basidiospores. Phylogenetic analysis indicates that *C. mucobrunneus* is nested in sect. *Defibulati* and is more closely related to *C. mucifluus* and *C. vanduzerensis* (Fig. 1). Morphologically, *C. mucobrunneus* showcases a dark brown pileus with tile red to platinum blonde margins alongside a white stipe, in contrast to *C. vanduzerensis* has a chestnut-black pileus with chestnut-brown margins and a light purple to dark lavender stipe (Smith and Trappe 1972). Furthermore, *C. mucobrunneus* has larger basidiospores [13–) 13.5–16 (–17) × 8–10 (–10.5) μm *vs*. (11–) 12–14 (–15) × 7–8 (–9) μm] than *C. vanduzerensis* (Smith and Trappe 1972). *Cortinarius mucobrunneus* is frequently misidentified as *C. mucifluus* due to the presence of translucent striations along the margin of the pileus, the pale stipe, and the cinnamon-hued annulus situated on the upper portion of the stipe. Nevertheless, *C. mucobrunneus* is characterized by a dark brown to liver brown pileus that exhibits broader basidiospores, in contrast to the ochraceous brown pileus of *C. mucifluus*, which features comparatively narrower basidiospores.

#### Cortinarius rugosiviscidus

Taxon classificationFungiAgaricalesCortinariaceae

T.S. Li, Y. Zhang & Q. Zhao sp. nov

E91B5A97-AA4C-563B-B816-82AB42A3D08C

860476

**Figure 3.**
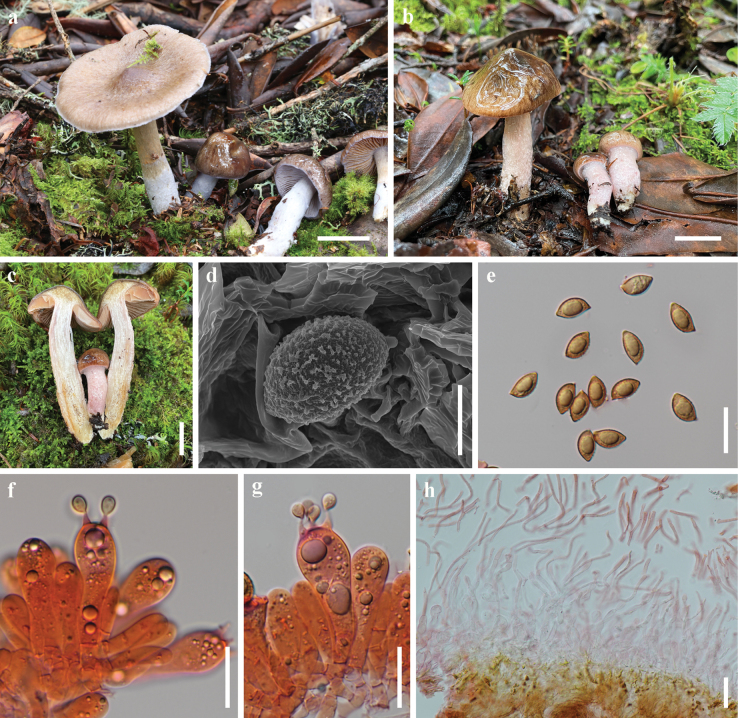
*Cortinarius rugosiviscidus*. a–c. Basidiomata; (a. HKAS 149458; b, c. HKAS 149457); d. Scanning electron micrograph of basidiospore; e. Basidiospores; f, g. Basidia; h. Pileipellis. Scale bars: 2 cm (a–c); 5 μm (d); 20 μm (e–g); 50 μm (h).

Fig. 3 Chinese name: 皱粘丝膜菌 (zhou nian si mo jun)

##### Etymology.

rugosiviscidus refers to the wrinkled and sticky surface of the pileus.

##### Holotype.

China • Yunnan Province: Lijiang City, Laojunshan Nature Reserve, at 26.631426°N, 99.719085°E, alt. 3960 m, in *Abies* forest, 25 Aug. 2018, LJ1634 (HKAS 149458).

##### Diagnosis.

Similar to *Cortinarius mucobrunneus* in the sticky pileus surface, but differing in the lighter pileus color and shorter basidiospores.

##### Macrostructures.

***Basidioma*** medium-sized. ***Pileus*** 20–65 mm diam., hemispherical, conical to campanulate, becoming applanate and umbonate with age; surface viscid, wrinkled when mature, dark blonde (5D4) to dark brown (6F8), becoming light brown (6C3) when mature; margin no radially striated; context 3–6 mm thick at mid-radius, white, then turns reddish blonde (5C4). ***Lamellae*** adnate to adnexed, 4–8 mm broad, moderately crowded, first light reddish golden (6C5), later brown (6E6); edge serrate. ***Stipe*** 40–100 × 8–15 mm, cylindrical, slightly curved, usually tapering upwards, solid; surface viscid, the upper part light brown (6C3), the lower part white; context white, sometimes with dark blonde (5D4) bruises. Basal mycelium white. ***Odour*** not distinctive.

##### Microstructures.

***Basidiospores*** [150/2/2] (11–) 11.5–15 (–15.5) × (7–) 7.5–9.5 (–10) μm [Q = (1.32–) 1.35–1.73 (–1.85), **Q** = 1.55 ± 0.08], elliptical to amygdaliform, brown in KOH, moderately verrucose. ***Basidia*** 34–55 × 12–18 μm, clavate, 4-spored. Marginal elements (n = 20) 19–30 × 9–12 μm, broadly fusiform, elliptical. ***Lamella trama*** hyphae 4–12 μm diam., smooth, colourless. Lamellar edges fertile. ***Pileipellis*** duplex: epicutis strongly gelatinous, 300–385 μm thick, composed of cylindrical, loosely arranged, long-celled, colourless hyphae 4–7 μm diam.; hypocutis 90–150 μm thick, composed of cylindrical to elliptical, strongly interwoven, colourless hyphae 5–14 μm diam., some hyphae with spiral or spotted encrustations. ***Stipe hyphae*** 3–8 μm diam., colourless, smooth. ***Clamp connections*** absent.

##### Habitat.

Mycorrhizal, gregarious in coniferous forests dominated by *Picea* and *Abies* trees.

##### Distribution.

Known from the southern Yunnan Province, China.

##### Additional material examined.

China • Yunnan Province: Lijiang City, Laojunshan Nature Reserve, at 26.631426°N, 99.719085°E, alt. 3960 m, in *Abies* forest, 10 Aug. 2023, ZY13 (HKAS 149457).

##### Notes.

*Cortinarius rugosiviscidus* is characterized by its victid basidiomata, light brown pileus with a wrinkled surface, brown lamellae, light brown stipe, and elliptical to amygdaliform basidiospores. Phylogenetic analysis indicates *C. rugosiviscidus* is nested in sect. *Defibulati* sister branch to *C. mucobrunneus* (Fig. 1). Morphologically, *C. rugosiviscidus* showcases a light brown pileus with a light brown stipe, in contrast to *C. mucobrunneus*, which has a dark brown pileus and a white stipe.

#### Cortinarius saloroides

Taxon classificationFungiAgaricalesCortinariaceae

T.S. Li, Y. Zhang & Q. Zhao sp. nov

D6F24E0B-5646-51D5-9482-3D77C8CDA263

860477

**Figure 4.**
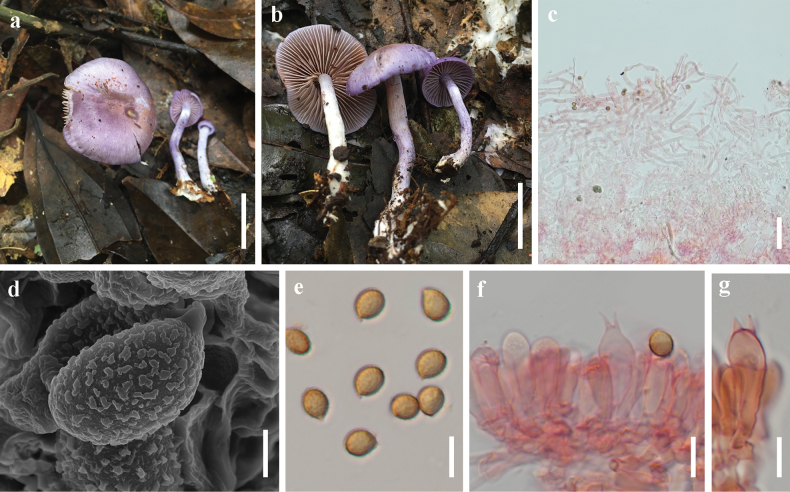
*Cortinarius saloroides*. a, b. Basidiomata (a. HKAS 149460; b. HKAS 149461); c. Pileipellis; d. Scanning electron micrograph of basidiospore; e. Basidiospores; f, g. Basidia. Scale bars: 2 cm (a, b); 50 μm (c); 2 μm (d); 10 μm (e–g).

Fig. 4 Chinese name: 类蓝紫丝膜菌 (lei lan zi si mo jun)

##### Etymology.

saloroides refers to species that are morphologically similar to *Cortinarius salor*.

##### Holotype.

China • Yunnan Province: Pingbian County, Daweishan National Forest Park, at 22.963949°N 103.702776°E, alt. 2250 m, in the rainforest, 15 Jul. 2020, PB94 (HKAS 149461).

##### Diagnosis.

Similar to *Cortinarius fibrillososalor* in the violaceous to whitish mauve pileus with fine fibrous scales, but differing in the thicker pileipellis.

##### Macrostructures.

***Basidioma*** small-sized. ***Pileus*** 15–45 mm diam., hemispherical to campanulate, becoming applanate and umbonate with age; surface dry, or viscid when wet, violet (17A8-17B8) to reddish violet (16B6), becoming mauve (17A5-17B5) when mature, with light brown (7D4) in the center and fine fibrous scales; margin inrolled when young, with remnants of universal veil; context 2–3 mm thick at mid-radius, white. ***Lamellae*** adnate to adnexed, 2–4 mm broad, moderately crowded, first white to mauve (17A5-17B5), later light brown (7D4); edge crenate. ***Stipe*** 20–50 × 4–6 mm, cylindrical, curved, usually tapering upwards; surface dry, bluish violet (18B6) or slightly lighter, leaving a light brown (7D4) ring on the upper part of the stipe; context white. Basal mycelium white. ***Odour*** not distinctive.

##### Microstructures.

***Basidiospores*** [100/2/2] 7–8.5 (–9) × (5–) 5.5–6.5 (–7) μm [Q = 1.11–1.38, **Q** = 1.24 ± 0.06], subspherical, broadly elliptical to elliptical, light brown to dark brown in KOH, moderately verrucose. ***Basidia*** 24–36 × 7–10 μm, clavate, 4-spored. ***Lamella trama*** hyphae 6–22 μm diam., smooth, colourless. Lamellar edges fertile. ***Pileipellis*** duplex: epicutis strongly gelatinous, 225–370 μm thick, composed of cylindrical, loosely arranged, long-celled, colourless hyphae 4–14 μm diam.; hypocutis 90–120 μm thick, composed of cylindrical to elliptical, strongly interwoven, colourless hyphae 8–23 μm diam., some hyphae with spiral or spotted encrustations. ***Stipe hyphae*** 3–10 μm diam., colourless, smooth. ***Clamp connections*** present. Cystidia absent.

##### Habitat.

Mycorrhizal, gregarious on the ground in tropical rainforests.

##### Distribution.

Known from the southern Yunnan Province, China.

##### Additional material examined.

China • Yunnan Province: Pingbian County, Daweishan National Forest Park, at 22.963949°N 103.702776°E, alt. 2250 m, in the rainforest, 29 Jul. 2019, PB-DWS 391 (HKAS 149460).

##### Notes.

*Cortinarius saloroides* is characterized by its violet pileus with fine fibrous scales, mauve to light brown lamellae, bluish violet stipe leaving a light brown ring on the upper part, and subspherical, broadly elliptical to elliptical basidiospores. Phylogenetic analysis indicates that *C. saloroides* is nested in sect. *Delibuti* sister branch to *C. fibrillososalor* (Fig. 1). Morphologically, *C. saloroides* showcases a violet pileus with fine fibrous scales on the surface and a bluish violet stipe, which can be confused with *C. fibrillososalor*. Nevertheless, *C. saloroides* can be distinguished from *C. fibrillososalor* by its thicker pileipellis, wider stipe hyphae, and slightly smaller basidiospores (Long et al. 2024).

## Discussion

Based on morphological characteristics and phylogenetic analysis of the ITS+LSU dataset, this study describes three new species of the genus *Cortinarius*, namely *C. mucobrunneus*, *C. rugosiviscidus*, and *C. saloroides*. In the phylogenetic framework, *C. saloroides* was positioned within sect. *Delibuti*, whereas *C. mucobrunneus* and *C. rugosiviscidus* were classified within sect. *Defibulati*.

Section *Delibuti* represents a highly diverse lineage, encompassing 16 newly described species in recent years (Ammirati 2018; Liimatainen and Niskanen 2021; Xie et al. 2021a; Bhunjun et al. 2022; Crous et al. 2022; Bojantchev 2023; Liu et al. 2024; Long et al. 2024; Siegel et al. 2024; Xie et al. 2025). The morphological characteristics of species within section *Delibuti* encompass a range of fruiting body colors, including blue, yellow, brown, or green; the pileus exhibits a sticky to viscous texture, while the lamellae exhibit a pale blue-purple hue that transitions to brown upon maturation. Additionally, mucus may be present, often accompanied by an annular zone located on the upper portion of the stipe. Basidiospores are observed to be nearly spherical to broadly elliptical in shape. (Peintner et al. 2004; Garnica et al. 2005; Soop et al. 2019). The newly described *Cortinarius saloroides* aligns morphologically with the characteristics of section *Delibuti* and is phylogenetically nested within this section. It forms a lineage with *C. fibrillososalor*, *C. sinosalor*, *C. subtropicus*, and *C. tibeticisalor*, which are characterized by violet pileus and are restricted to East Asia, consistent with Long et al. (2024). *Cortinarius fibrillososalor* can be well distinguished from other species in the sect. *Delibuti* by the fibrils on the surface of the pileus (Long et al. 2024). It is worth noting that *C. saloroides* exhibits a similar trait, making it challenging to differentiate from *C. fibrillososalor* by macroscopic morphology. Currently, within the sect. *Delibuti*, two species are identified that possess a violet pileus and fibrillose surface texture. Nonetheless, these species can be distinguished by the thickness of the pileipellis, the width of the stipe hyphae, and the length of the basidiospores. Furthermore, BLAST analysis has indicated that the ITS sequence of *Cortinarius saloroides* shows 92.51% similarity to that of the type specimen of *C. fibrillososalor*, with 53 nucleotide substitutions and insertion-deletion (indel) positions.

In sect. *Defibulati*, species exhibit consistent morphological characteristics, mainly including: the pileus and stipe covered with mucus, relatively large elliptical to amygdaliform basidiospores with warty surfaces, the absence of clamp connections, and incomplete development of the veil, among other features (Ammirati et al. 2007; Soop et al. 2021). Additionally, ITS sequences of species in this section show minimal variation, differing by only 2–3 base sites (Seidl 2000). The other two new species described in this study, *Cortinarius mucobrunneus* and *C. rugosiviscidus*, are nested in sect. *Defibulati* based on phylogenetic analysis. They form sister clades and are closely related to *C. mucifluus* and *C. vanduzerensis*. Morphologically, the stipe of *C. vanduzerensis* is light purple to dark lavender, differing from the white stipe of *C. mucobrunneus* and the brownish stipe of *C. rugosiviscidus*. The pileus of *Cortinarius rugosiviscidus* is dark blonde to dark brown, contrasting with the ochraceous brown pileus of *C. mucifluus*. BLAST analysis showed that the ITS sequence of *C. mucobrunneus* is most similar to that of *C. vanduzerensis* (FJ717562), with 98.65% similarity and 12 substitutions/indels. Similarly, the ITS sequence of *C. rugosiviscidus* is closely related to *C. vanduzerensis* (FJ717562), showing 98.38% similarity and 13 substitutions/indels. A total of 8 substitutions/indels were observed between the ITS sequences of the type specimens of *C. mucobrunneus* and *C. rugosiviscidus*. Both morphological and molecular evidence confirm that *C. mucobrunneus* and *C. rugosiviscidus* are distinct, independent species within sect. *Defibulati*.

*Cortinarius* has established itself as a paramount taxonomic clade in mycology, distinguished by its remarkable species diversity, essential ecological roles, and complex classification structure. Advances in molecular systematics have significantly enhanced our understanding of the genus’s classification, phylogenetic relationships, and associated ecological functions (Wang et al. 2025). Historically, species identification was predominantly based on morphological traits prior to the incorporation of molecular data. In China, where research in fungal taxonomy has lagged behind that of other regions, numerous species within the genus *Cortinarius* have been inaccurately classified alongside their European or American counterparts (Xie et al. 2023). As it is renowned for possessing one of the most diverse fungal ecosystems worldwide, particularly within biodiversity hotspots like Yunnan, China, it harbours a significant array of endemic and unique *Cortinarius* species. However, historical constraints in research methodologies have hindered a thorough elucidation of the taxonomic status and true diversity of these taxa.

## Supplementary Material

XML Treatment for Cortinarius mucobrunneus

XML Treatment for Cortinarius rugosiviscidus

XML Treatment for Cortinarius saloroides

## Additional information

### Use of AI

No use of AI was reported.

### Author ORCIDs

Tai-Shun Li https://orcid.org/0000-0001-8697-1630

Hong-Bin Guan https://orcid.org/0009-0007-4113-2615

Yan-Yan Yang https://orcid.org/0009-0007-9521-558X

Fang Wu https://orcid.org/0000-0002-1455-6486

Qi Zhao https://orcid.org/0000-0001-8169-0573

Ying Zhang https://orcid.org/0009-0000-5248-0171

### Data availability

All of the data that support the findings of this study are available in the main text.
